# Development of an online curriculum for California early care and education providers on healthy beverages

**DOI:** 10.1186/s12889-021-11428-x

**Published:** 2021-07-13

**Authors:** Kimberly Hazard, Danielle Lee, Lorrene Ritchie, Roberta Rose, L. Karina Díaz Rios, Kaela Plank, Abbey Alkon

**Affiliations:** 1 University of California, San Francisco (UCSF) School of Nursing, California Childcare Health Program, 2 Koret Way, Box 0606, San Francisco, CA 94143-0606 USA; 2 University of California, Division of Agriculture and Natural Resources, Nutrition Policy Institute, 2115 Milvia Street, 3rd Floor, Berkeley, CA 94704 USA; 3 University of California, Merced, Division of Agriculture and Natural Resources, 5200 North Lake Road, Merced, CA 95343 USA

**Keywords:** Online training, Child care, Early care and education, Professional development, Healthy beverages, Sugar-sweetened beverages, Childhood obesity

## Abstract

**Background:**

Children’s consumption of sugar-sweetened beverages is associated with obesity, diabetes, and dental decay. California’s Healthy Beverages in Child Care Act (AB 2084) requires all licensed child care centers and family child care homes to comply with healthy beverages standards, however many licensed providers in California are unaware of the law and few are fully compliant with the law’s requirements. The aim of the current project is to describe the development of a self-paced online training on best practices and implementation of AB 2084 in English and Spanish for family child care home and child care center providers; and to evaluate the feasibility, defined as being accessible, acceptable, and satisfactory to providers, of this new online course.

**Methods:**

The project was broken into two main stages: (1) development of the online course; and (2) evaluation of the final online course. The first stage was completed in five phases: (1) identify relevant course content and develop narration script; (2) conduct in-person focus groups with child care providers to review and edit the content; (3) adapt course content and translate for Spanish-speaking providers; (4) build the online course and resources; and (5) pilot online course and evaluate accessibility. The second stage, evaluation of the acceptability and satisfaction of the final course was rated on a Likert scale from 1 to 4; the evaluation was completed as part of a larger randomized control trial with 43 child care providers. The course features four key requirements of AB 2084 as the main sections of the course (milk, sweetened beverages, juice, and water), plus background information about beverages and children’s health, special topics including caring for children with special needs, family engagement, written policies, and child engagement.

**Results:**

The child care providers who completed the evaluation found the online training was easily understandable (median(Q1,Q3,IQR) = 4 (4,4,0)), included new information (3 (1, 3, 4)), provided useful resources (4(4,4,0)), and was rated with high overall satisfaction (3 (1, 3, 4)).

**Conclusion:**

Online training in English and Spanish designed for child care providers is a feasible medium to deliver important health messages to child care providers in an accessible, acceptable, and satisfactory manner.

**Supplementary Information:**

The online version contains supplementary material available at 10.1186/s12889-021-11428-x.

## Introduction

There is substantial evidence that young children who consume sugar-sweetened beverages have an increased risk of childhood overweight/obesity and dental caries [1]. In the United State (U.S.), the prevalence of obesity was 13.9% among 2- to 5-year-olds and 18.4% among 6- to 11-year-olds in 2015–2016 [2]. To address the high prevalence of obesity, California passed the Healthy Beverages in Child Care Act (AB 2084), which was implemented in 2012 [3]. This law requires all licensed child care facilities to make clean and safe drinking water readily available and accessible for children to drink throughout the day, serve no more than one serving (4 oz / 0.11 kg) per day of 100% juice, serve only unflavored, low-fat (1%) milk or nonfat (skim) milk to children two years of age or older, and prohibits serving beverages with added natural or artificial sweeteners. Another new California law, AB 290, requires all newly licensed California child care providers to receive one hour of in-person nutrition education starting January 2016 [4]. This provided a mechanism to disseminate information on AB 2084, however, child care programs licensed before 2016 may not have the same awareness of the healthy beverages law.

Over a year after AB 2084 was passed into law, a cross-sectional survey was conducted of licensed child care providers in California. Only 60% of participating child care providers (*n* = 435) were aware of the law and only about one quarter were fully compliant with the law’s requirements [5, 6]. Four years later, another cross-sectional survey of licensed child care providers was conducted, and found that only 45% of these providers were fully compliant with AB 2084 [7]. California child care providers and stakeholders expressed the need for provider training to identify the potential challenges associated with serving healthy beverages and developing strategies to support healthy beverages in child care programs [5].

Regulations focused on increasing young children’s intake of healthy beverages in child care programs are not unique to California. Child care programs participating in the U.S. Department of Agriculture (USDA) Child and Adult Care Food Program (CACFP) must meet similar federal beverage requirements as California’s child care programs under AB 2084, including requirements for drinking water to be offered and available upon request throughout the day, prohibiting flavored milk for children ages 2 through 5, and limiting service of juice to once per day [8, 9]. In many states, including California, all licensed child care centers are required to meet CACFP nutrition standards even if they do not receive federal funding, and several states have laws similar to AB 2084 that require that child care providers offer specific beverages to the children in their care [10]. For example, child care center licensing regulations in Arizona, Colorado, Illinois, Maryland, Mississippi, New Jersey, New York, North Carolina, Oklahoma, and Rhode Island prohibit sugar-sweetened beverages to be served in their child care centers.

In-person trainings for child care providers on healthy beverage practices have been shown to improve children’s consumption of healthy beverages [11]. Although in-person trainings have a positive impact on child care providers, child care providers are often unable to attend in-person trainings. An Institute of Medicine (IOM) and National Research Council (NRC) report called for improved early care and education (ECE) workforce development and listed several barriers to ECE providers gaining professional development: lack of time, lack of funds, lack of a professional community, high staff turnover, the need to constantly retrain, and lack of availability of professional learning activities [12].

Studies of online trainings for Child Care Health Consultants and teachers have demonstrated positive knowledge change comparable to that of in-person instruction [13, 14]. Findings from a study of a child sexual abuse prevention program for child care providers indicated that both in-person and online training formats were feasible to implement and acceptable to them [15]. The findings from online trainings among child care providers [16] and Special Supplemental Nutrition Program for Women, Infants, and Children participants [17, 18] show that online nutrition trainings can be a culturally acceptable, effective, and cost-efficient way to disseminate nutrition education. There are online trainings available for child care providers in some states (New York, Pennsylvania, and Washington) that include information on healthy beverages, but these trainings are not available in Spanish, and are not limited to 30 min or less, and have not been formally evaluated.

As online training options expand for child care providers, it is important to evaluate the trainings to learn if they are accessible, acceptable, and satisfactory for the child care providers working in both child care centers and family child care homes. To address these gaps in the field, we developed and evaluated an online training on healthy beverages for child care center and family child care home providers. Additionally, about 30% of the population in California speak Spanish at home and 13% have limited ability to speak English [19]. Over half of center-based child care providers in California speak a language other than English [20]. Therefore, online professional trainings should be developed for child care providers in English and Spanish. This paper reports on the development of a self-paced, online training on best practices for children’s healthy beverage consumption in child care programs, available in English or Spanish; and evaluation of the feasibility, defined as being accessible, acceptable, and satisfactory to providers of the final online training.

## Methods

The development, design, and evaluation of the online training was part of a larger study led by the Nutrition Policy Institute (NPI) at the University of California Division of Agriculture and Natural Resources (UC ANR) to evaluate the training using a randomized control trial (RCT). The interdisciplinary study team included nutritionists, nurses, educators, child care providers, and consultants. The University of California, San Francisco (UCSF) School of Nursing, California Childcare Health Program (CCHP) led the development phase of the online course, including the focus group, and NPI led the pilot and RCT evaluation.

The UCSF-CCHP project team established guidelines for the online course to engage the child care provider user more than reading text or listening to lectures alone, to visually demonstrate strategies for implementation, and to be appropriate for a wide range of literacy levels. These guidelines were that the course would take no longer than 30 min to complete, incorporate interactive quizzes/games, include primarily voice-over (narration), and utilize videos.

The project was broken into two main stages: [1] development of the online course; and [2] evaluation of the final online course. The first stage was completed in five phases: [1] identify relevant course content and develop narration script [2]; conduct in-person focus groups with child care providers to review and edit the content [3]; adapt course content and translate for Spanish-speaking providers [4]; build the online course and resources; and [5] pilot online course and evaluate accessibility. The second stage, evaluation of the acceptability and satisfaction of the final course, was led by NPI as part of the RCT with 43 child care providers, with results presented below.

### Stage 1: development of the online course

#### Phase I: identify content and develop narration script

The course content was developed using the summary of meetings held with UCSF-CCHP project staff, NPI, and national experts on drinking water and children’s health. An outline of the course content was developed and included the four key requirements of the AB 2084 as the main sections of the course, plus research about beverages and children’s health, special topics including caring for children with special needs, family engagement, written policies, and child engagement. To avoid duplication of available online training on serving healthy beverages to children, existing online courses (from New York State’s Office of Children and Family Services, PennState Extension, and The Washington State Department of Children, Youth, and Families) and stand-alone resources (i.e., handouts, educational materials) were compared for developing the script and additional resources. These online trainings were not available in Spanish, often exceeded 30 min, and had not been formally evaluated.

UCSF-CCHP staff developed slides as a draft of the course content, serving as a template for the online course’s screen display, accompanied by a script for what would later be the online course’s voice-over narration. The first draft of slides and narration script were critically reviewed by project staff and consultants with expertise on beverages for young children to ensure the accuracy of the content. UCSF-CCHP staff incorporated the expert feedback on both the visible slide text and narration script. The resulting version of the course was further edited by incorporating focus group feedback.

#### Phase II: focus group

To determine the relevance and content of the course, UCSF-CCHP organized a focus group of child care providers to attend a slide presentation of the course content. The focus group questions and protocol were developed by UCSF-CCHP and reviewed by NPI and the team of expert advisors. The focus group script and protocol, demographic survey, and consent form were reviewed and approved by the Institutional Review Board at the University of California, San Francisco. The focus group was recorded and notes were taken by a UCSF-CCHP project staff member. Providers received a gift card for their participation. Eight English-speaking, female child care providers from both family child care and centers attended the two-hour focus group. Of the eight providers, two were Hispanic / Latino, two were Non-Hispanic White, three were African American, and one was Asian; three participated in CACFP; seven cared for children that receive child care subsidies; and all had at least some college education.

After signing consent forms and completing an icebreaker exercise, focus group participants listened to the slide presentation of the healthy beverages course. The slides were presented by UCSF-CCHP staff with scripted narration and they facilitated a discussion session with six open-ended questions. The questions covered different domains, including content covered, additional content to incorporate, and mode of delivery (questions and participant responses summarized in Table 1). At the close of the focus group discussion, the facilitators then summarized the key points of the discussion and asked the participants to confirm the accuracy of the summary.

**Table 1:** Focus group results: Themes and modifications to course

Focus group question	Response themes	Modifications made to course
What information is new to you? What information is helpful? What information is too repetitive?	- There is a wide range of baseline knowledge. - Providers who participate in CACFP and newer providers are more familiar with this content.	- Incorporated ideas for implementing best practices, rather than just sharing information alone.
Is there something you would like to know more about?	- Milk: Differences between organic and hormone-free; allergies and intolerances; and nutritional equivalents - Juice: reading labels, making home-made juice, serving size, concentrates vs. non-concentrated - Water: bottled water quality, fluoride safety, tap water contaminants - Non-caloric and artificial sweeteners added sweeteners such as stevia okay? - Child engagement: involving children is important	- Developed a Health and Safety Note^1^ on milk that addresses these questions to include with resources. - Addressed these juice topics in narration and added photos of juice labels and illustrated what to look for. - Developed a Health and Safety Note on water that addresses these questions to include with resources. - Addressed question about non-caloric and artificial sweeteners in narration. - Added more narration about child engagement and developed handout on child engagement and water for the resources.
Would you take this training? What could be improved?	- There is more need for parent education than provider education. Allow parents to take final training. - 15 min would be the ideal length. - Make sure it is interactive and keeps people’s attention.	- Added more ideas for family engagement. - Attempted to shorten the length of the course but were unable to include all the necessary information in under 15 min. - Added interactive games and quizzes and developed the “Dewey the Droplet” character.

^1^*Health and Safety Notes* are written for professionals working in the field of early care and education (ECE). They focus on issues that frequently arise in ECE, such as how to handle a child who bites; specific illnesses and diseases; and best practices for cleaning, sanitizing, and disinfecting.

A UCSF-CCHP staff member transcribed the recorded focus group and another staff member conducted a thematic analysis to identify common topics, ideas, and patterns of meaning (see response themes in Table 1). The thematic analysis informed the next phase of modification and resource development. The updated content was reviewed by NPI and the team of expert advisers to validate the course content to be used in the built online training. Some of the updated content included child and family engagement.

#### Phase III: language adaptation

The slide content and narration script were translated into Spanish by professional services at the UC. A bilingual, native Spanish speaking registered dietitian with expertise in cultural adaptation verified the translation for equivalence to the English version. The verified version was used in two rounds of one-on-one in-depth interviews with a total of four Spanish-speaking providers to ascertain: a) the clarity and familiarity with the terminology and overall language, b) the cultural relevance of the content, and c) the perceived acceptability of the online format (see Supplementary Table 1). Interviews were conducted by the same researcher who verified the content equivalence.

In the first round of interviews, two providers identified five words as unknown and six unfamiliar terms and suggested alternative language to replace them. This initial version was then modified to incorporate providers’ feedback and then presented to two additional providers to confirm the adequacy of changes (Supplementary Table 2). No language issues were flagged in the second round of interviews. All four providers said the script was easy to follow, that the information was relevant, and deemed the online format as appropriate and convenient.

#### Phase IV: build online course and resources

**Fig. 1:**
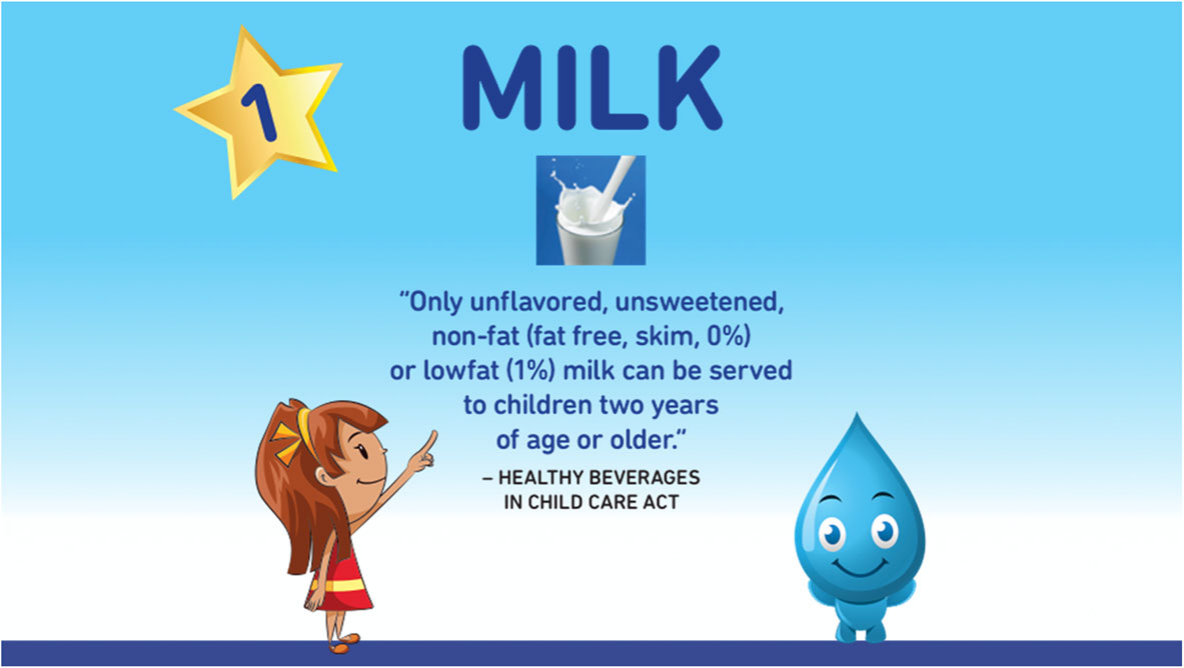
Image of slide introducing first main section, featuring “Dewey the Droplet” in English. Slide and images developed by UCSF-CCHP in partnership with NPI, 2017

**Fig. 2:**
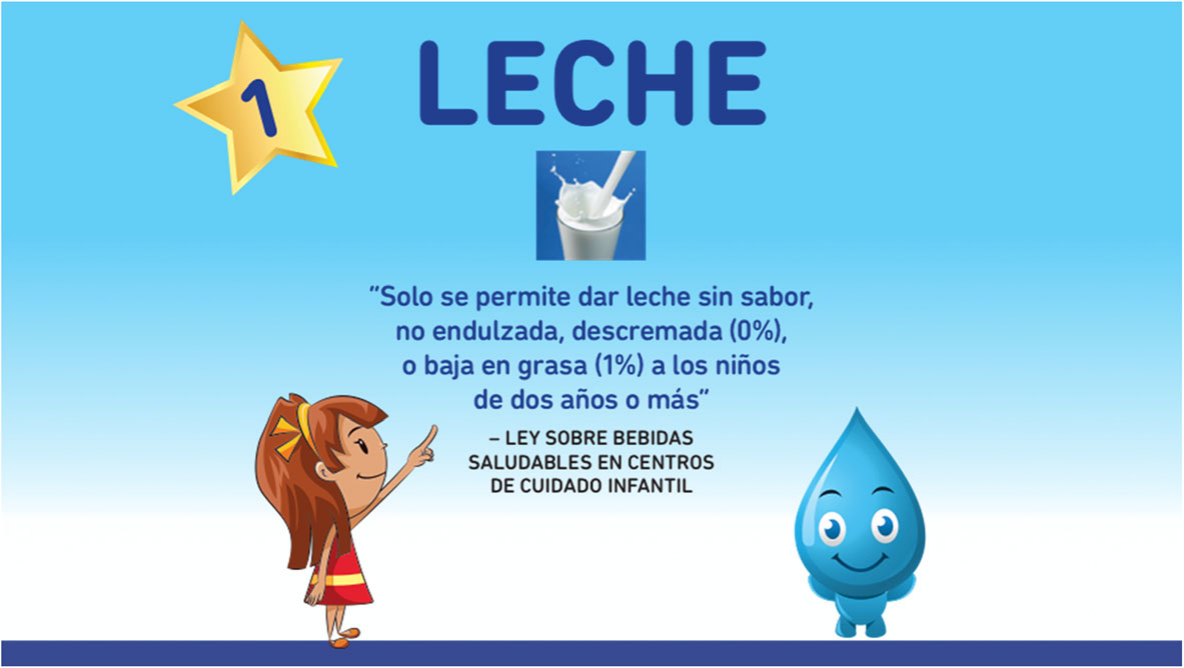
Image of slide introducing first main section, featuring “Dewey the Droplet” in Spanish. Slide and images developed by UCSF-CCHP in partnership with NPI, 2017

The online course was built in Articulate Studio™ (Articulate, New York, NY). A graphic designer created the slide deck design and illustrated the character, “Dewey the Droplet” (Fig. 1 and Fig. 2). The illustration was included to add interest in the form of a child-friendly character and to reinforce key points. The formatted slides were then put into Articulate Studio™ and two UCSF-CCHP staff provided the English narration, alternating narration by each major section of the course. Spanish translators provided narration at the UCSF computer lab. Three interactive games were created in Articulate Studio™ by UCSF-CCHP staff. Pediatric Nurse Practitioner masters’ students developed a short video to show how water can be made available and accessible, indoors and outdoors, at meals and at snacks, throughout the day at child care centers or homes. UC video services staff helped finalize the video and translation services provided narration for the Spanish version. UCSF-CCHP developed additional Health and Safety Notes on topics that needed concise information sheets where resources did not already exist.

UCSF-CCHP staff edited the audio and added music to the opening and closing credits before finalizing and packaging the final course on Articulate Studio™ (see Table 2). Although the goal was to develop a course that was 30 min or less, the final running time for the English course was 29 min and the Spanish course was 37 min. The course could be paused and completed over more than one sitting, and was broken up into short segments by topic (see Table 2). A Flesch-Kincaid readability test was used to indicate how easy or difficult the English text was to understand. The Flesch–Kincaid Grade Level Formula presents a score as a U.S. grade level. The final narration script achieved a 5.5 grade level.

**Table 2:** Final course topics and features

Course Topic	Online Feature (in addition to slide text and voice-over narration)
Overview and Introduction - Beverages and children’s health - Laws and regulations	- Annotation to show users where to access resources, glossary, and table of contents
Message #1: Milk	- Simulated text message segment - Quiz: select correct types of milk by clicking images (animated screen depending on correct or incorrect answer)
Message #2: No Sweetened Beverages	- Annotation of sample ingredient label - Quiz: select which beverages have added sweeteners by clicking images (animated screen depending on correct or incorrect answer)
Message #3: 100% Juice	- Annotation of sample ingredient label
Message #4: Water	- Embedded video
Summary and Additional Topics - Children with Special Needs - Family Engagement - Written Policies - Ideas for Curriculum	- Quiz: puzzle matching beverage with how often it should be served
Resources	- Button on left side of viewer with drop down of documents and links
Glossary	- Tab that can be accessed while viewing the training

#### Phase V: pilot online course to assess accessibility

Once the English and Spanish courses were finalized in Articulate Studio™, they were uploaded to the learning management system, Moodle Classroom Learning Environment (CLE). UC ANR hosts other online courses on eXtension Foundations’s CLE, called Campus (English: https://campus.extension.org/enrol/index.php?id=1722, Spanish: https://campus.extension.org/enrol/index.php?id=1739&lang=es). The protocols and consents for participating in the pilot and RCT were reviewed and approved by the University of California, Davis Institutional Review Board.

The course hosted on ANR’s CLE platform (http://class.ucanr.edu) was taken by seven child care providers from three Northern California counties. The providers worked in either child care centers or family child care homes and took the online course on mobile phones or computers with various operating systems. Five of the pilot participants took the course in English and two participants took it in Spanish.

The pilot participants provided both positive and negative feedback on accessing the course. Most of the providers stated that the course was easy or very easy to navigate, and most of the providers were able to start or stop the course at a pace that was right for them. The participants commented that they enjoyed the format and information provided; thought the interactive quizzes were motivating, helped them pay attention, reinforced the information presented; the Spanish version was clear with appropriate translations; and the voice-over and word choices were clear, simple, and understandable.

The providers also identified challenges including registration and accessing resources. In response to this feedback changes were made to the course interface, such as editing registration instructions and giving providers an enrollment key rather than registering for the course under the CLE system. The resources, glossary, and the ‘Certificate of Completion’ were re-configured to make them easier to access, appearing on the CLE landing page, rather than embedding them within the online course. Additionally, steps were taken to make the course compliant with The Americans with Disabilities Act (ADA), including providing a written transcript for audio files and closed captioning for the embedded video. Since the provider feedback focused on technical logistics or administrative issues, no changes were made to the content of the course.

### Stage 2: evaluation of the final online course

The online course was evaluated using a survey filled out online or on paper by participants who completed the course as part of the larger RCT study. Licensed child care centers and family child care home providers from California’s Central Coast, San Francisco Bay Area and Central Valley regions were recruited through Resource and Referral Agencies and local child care networks using recruitment materials available in both English and Spanish. Child care providers (*n* = 43, see Table 3) that cared for children ages 2–5 years, provided beverages during at least one meal or snack, and not at a Head Start or State preschool were enrolled in the study to take the online course. Providers completed a brief online (© 2020 Qualtrics®) survey regarding their experience with the online course – including acceptability of the length, clarity, content, and usefulness; and satisfaction with the different components of the training. Participating providers received the survey via email or text message; those that did not complete the online survey within 7 days were mailed a paper survey which they returned via a pre-paid self-addressed envelope. Upon completing the online course and survey, providers were mailed a gift card. The child care providers were asked to rate how much they agreed or disagreed with statements about the training content novelty, understandability, length, interest level, and resource usefulness on a Likert-scale from 1 to 4 (1 = Disagree a lot, 2 = Disagree a little, 3 = Agree a little, 4 = Agree a lot (see Table 4). Likert scale survey responses were treated as continuous variables and descriptive analyses were conducted. All item level outcome variables were tested for normality using the Shapiro-Wilks Test (*p* < 0.05 was considered non-normal) and were visually evaluated using histograms. Differences in Likert-scale responses to survey questions by child care type (center-based vs. family child care) were compared using Wilcoxon Mann Whitney tests. Significance was set at *p* < 0.05. Due to the small sample size of Spanish-only speaking providers enrolled in the evaluation study (*n* = 2), differences in online training experiences could not be assessed between English-speaking and Spanish-speaking providers. Analyses were conducted in SAS 9.4 (SAS Institute, Cary, NC).

**Table 3:** Demographic characteristics of the RCT participants

Characteristic		Participating child care providers (*n* = 43) N (%)
Sex	Female	42 (98%)
Race / Ethnicity	African American	4 (9%)
	Asian	7 (16%)
	Hispanic / Latino	9 (21%)
	Non-Hispanic White	21 (49%)
	Other	2 (5%)
Age	21–30	3 (7%)
	31–40	8 (19%)
	41–50	13 (30%)
	Over 50	19 (44%)
Education	Some college / AA	23 (54%)
	Bachelor’s Degree	20 (47%)
Preferred Language	English	41 (95%)
	Spanish	2 (5%)
Child Care Setting	Child Care Center	20 (47%)
	Family Child Care Home	23 (53%)
CACFP Participation	Yes	20 (47%)
	No	21 (49%)
	Don’t Know	2 (5%)
Care for Children with Subsidies	Yes	26 (60%)
	No	17 (40%)

**Table 4:** Average Likert-scale* response from providers level of agreement with statements about the online course acceptability

Statement	Middle Quartile (Median) *n* = 43	Lower Quartile (Q1)	Upper Quartile (Q3)	Inter-Quartile Range (IQR)
I learned something in the online training that I did not know before.	3	3	4	1
I understood everything in the online training	4	4	4	0
The training was too long.	2	1	2	1
The training could be more interesting.	2	2	3	1
The resources provided with the training were useful.	4	4	4	0

*Scored from 1 to 4 with 1 = Disagree a lot, 2 = Disagree a little, 3 = Agree a little, 4 = Agree a lot

## Results: evaluation of course acceptability and satisfaction

The item level tests for normality were statistically significant (*p* < 0.05) so non-parametric statistics were used for the subsequent analyses. Providers strongly agreed that the course was understandable (median(Q1,Q3, IQR) = 4(4,4,0)) and that the resources provided were useful (4(4,4,0)). Providers agreed that they learned something new in the course (3 [1, 3, 4]). Providers disagreed with the statement that the course was too long (2 [1, 2]) and that the course could be more interesting (2 [1–3]). No significant differences were found between center and family child care providers on measures of satisfaction and acceptability.

The CLE administrators were able to track the number of providers who accessed the resources available as part of the course: the resource list document, the glossary of terms, the full packet of curriculum slides, and the certificate of completion. 54% of the providers accessed the resource list document.

The median(Q1,Q3,IQR) overall satisfaction with the online training among the 43 providers that completed the online training was 3 [1, 3, 4] on a Likert-scale of one to four (Table 5). Each major content section scored similarly with a median of 3 and a Q1, Q3, and IQR of 3,4 and 1 for all sections. No significant differences in satisfaction were found between center and family child care providers.

**Table 5:** Mean Likert-scale* response from providers on satisfaction with different components of the training

Training component	Middle Quartile (Median) *n* = 43	Lower Quartile (Q1)	Upper Quartile (Q3)	Inter-Quartile Range (IQR)
Interactive Games	3	3	4	1
Milk Section	3	3	4	1
Sweetened Beverages Section	3	3	4	1
100% Juice Section	3	3	4	1
Water Section	3	3	4	1
Overall Satisfaction	3	3	4	1

* Scored from 1 to 4 with 1 = Very Unsatisfied, 2 = Unsatisfied, 3 = Satisfied, 4 = Very Satisfied

## Discussion

The purpose of this study was to describe the development of a self-paced online training on healthy beverage best practices in child care programs, available in English and Spanish; and to evaluate the accessibility, acceptability, and satisfaction of the online training. In the first stage of the project, we developed and modified the course using feedback from focus groups and a pilot study of child care providers, resulting in the final online training. We aimed for a course that was concise, included voice-over narration and videos, and incorporated interactive quizzes/games, as identified in Cotwright et al. as the preferred eLearning format for an online beverage policy training for child care providers [16]. The child care providers who participated in the development of the course found the online training accessible and acceptable in both English and Spanish. One shortcoming of the final course was that the Spanish version exceeded our desired length of 30 min. However, the course was not required to be completed at one time and was divided into six segments and featured three interactive games. These short segments and interactive elements are consistent with adult learner literature suggesting a maximum of 20-min learning sessions [21]. In addition, the surveyed providers did not express concern about the training length for either the English or Spanish versions.

In Stage 2 of the project, we evaluated the final online course as part of a larger RCT study. These child care providers found the online training acceptable and satisfactory, as found in other studies of online trainings for child care providers, on nutrition education, or both [15–18, 22]. The comments from focus group participants and the ratings by evaluation participants in the present study suggest that developing an online training that is useful and interesting for a wide range of child care providers is feasible and acceptable. Because child care providers participating in CACFP have monitoring and technical assistance provided by the state or local agencies on nutrition standards [23], they likely have more experience with the beverage policy. We incorporated ideas for implementing healthy beverage best practices, rather than information on beverage requirements alone.

Our evaluation of the course does have limitations. We did not assess the child care providers’ previous exposure to online training courses or overall comfort with technology. According to the National Center for Education Statistics, adults who are not digitally literate are, on average, have fewer years of education, are older, and more likely to be Black, Hispanic, or foreign born, compared to digitally literate adults [24]. We hypothesize that providers with less exposure to web-based material may require additional guidance to access and complete the training; and that the online format might be more acceptable to providers with previous experience taking online trainings. Our convenience sample of child care providers may over-represent providers who seek new opportunities and engage in online trainings. Finally, although we conducted the evaluation for both English and Spanish providers, we did not develop different questions to assess cultural relevance or literacy. Evaluation study participants were largely English speaking, and only two participants opted to take the Spanish version. Evaluating the online training with a larger group of Spanish-only speaking providers is necessary to ensure the training is well accepted by this group of providers.

While online trainings offer the benefit of convenient access and lower cost [25, 26], we experienced several challenges. Technology rapidly changes and some platforms for hosting or developing online training may change. During our development of the training, the Articulate Studio™ desktop software moved to a cloud-based platform, Articulate 360™ and the original files became outdated before launching the larger intervention study, which delayed the start date of the RCT. Further, once ANR switched from hosting online trainings on their own site (class.ucanr.edu) to an external collaborator (campus.extension.org), which utilized a newer version of Moodle learning management system (LMS), the online training had to be modified once again in Articulate 360 to ensure the output file (known as a SCORM – shareable content object reference model) would work in the new LMS.

## Conclusion

The development of an online curriculum for child care providers to identify and serve healthy beverages in early care and education programs was shown to be an efficient and acceptable method for providing health and safety training to child care providers. When considering the many barriers that child care providers face to attending in-person health and safety trainings, including lack of substitute caregivers or financial support for professional development, having the option to complete a self-paced, free or low-cost online training is appealing. Offering online trainings may be advantageous to the agencies that provide trainings to child care providers as well, eliminating the cost associated with in-person trainings, including hiring one or several expert trainers. However, considering the varying levels of digital literacy and technological skill among child care providers and the changes in technology, care should be taken to provide someone equipped to answer questions and help trouble-shoot at the agency level.

This online training can serve as a model for the specific content – healthy beverages in ECE – and, more broadly, as a promising medium for delivering health and safety training for child care professionals. Current trends indicate increasing adoption of digital technologies in the workplace, including for workforce training [27]. This course focused on California-specific content; however the best practices around healthy beverages in child care apply nationwide and the course content could be easily adapted to other states’ needs. Overall, online training in English and Spanish designed for child care providers is a feasible medium to deliver important health messages to child care providers in a manner they find accessible, acceptable, and satisfactory.

## Supplementary Information

**Additional file 1 Supplementary Table 1**. Interview Questions for Spanish Speaking Providers. **Supplementary Table 2**. Spanish-Speaking Provider Interviews: Flagged Language and Suggested Replacements.

## Abbreviations

ADA: Americans with Disabilities Act
CACFP: Child and Adult Care Food Program
CCHP: California Childcare Health Program
CLE: Classroom learning environment
ECE: Early care and education
IOM: Institute of Medicine
LMS: Learning management system
NPI: Nutrition Policy Institute
NRC: National Resources Council
UC ANR: University of California Division of Agriculture and Natural Resources
UCSF: University of California, San Francisco
