# Supplementary material for: Development of an online curriculum for California early care and education providers on healthy beverages
Source: BMC Public Health. 2021 Jul 13;21:1387. doi: 10.1186/s12889-021-11428-x (PMC8276417; doi:10.1186/s12889-021-11428-x)
Supplement: Supplementary file 1 — Additional file 1 Supplementary Table 1. Interview Questions for Spanish Speaking Providers. Supplementary Table 2. Spanish-Speaking Provider Interviews: Flagged Language and Suggested Replacements. [file 12889_2021_11428_MOESM1_ESM.docx]

**Supplementary Tables**

**Supplementary Table 1. Interview Questions for Spanish Speaking Providers.**

| 1. Do you think the words used are clear? 2. Are there any words or phrases you would say differently? 3. Do you think other providers who speak Spanish can follow the narration easily? 4. Is there any information you believe is particularly important or relevant to Spanish-speaking providers? 5. Is there any information that you think may be particularly problematic for Spanish-speaking providers? 6. Is there something you think Spanish-speaking providers would like to know more about regarding healthy beverages? 7. How comfortable are you with the idea of taking an online training? 8. Do you think other Spanish-speaking providers would feel comfortable taking a training online? 9. If you were offered the opportunity, would you take this training? Why or why not? |
| --- |

**Supplementary Table 2. Spanish-Speaking Provider Interviews: Flagged Language and Suggested Replacements.**

**Unknown words** – participant does not recognize the word or hasn’t heard it before:

| **Spanish** | **English** | **Replacement suggestions** |
| --- | --- | --- |
| *Edulcorante* | Sweetener | *Endulzante* |
| *Desnatado* | Skim | *Descremado*  *Sin grasa* |
| *Macedonia* | Fruit salad | *Cóctel de fruta*  *Ensalada de frutas* |
| *Pinchos o brochetas* | Fruit kabobs | *Fruta picada* |
| *Escorrentías* | Farm runoff | *Drenaje agrícola* |

**Unfamiliar words** – participant recognizes meaning but prefers/suggests using another word:

| **Spanish** | **English** | **More familiar suggestions** |
| --- | --- | --- |
| *Bebida de jugo*  *Coctel de jugo*  *Ponche de jugo* | Fruit drink  Juice cocktail  Fruit punch | *Bebida de fruta*  *Coctel de frutas*  *Ponche de frutas* |
| *Fruta troceada* | Chopped fruit | *Fruta en trozos*  *Fruta picada* |
| *Agua del grifo* | Tap water | *Agua de la llave* |
| *Meriendas en los que todos participen* (potluck) | Potluck | *Convivio* |
